# Dr. H. J. McKellops

**Published:** 1901-05-15

**Authors:** 


### OBITUARY.

### DR. H. J. McKELLOPS.

Dr. H. J. McKellops, one of our best known dentists, died April 23rd at his home in St. Louis, Mo. He was never a scientist, but did much to stimulate scientific research. He was greatly interested in the profession's literature, and probably possessed the most complete private dental library in existence. He gave much time and thought to the development of the technical branch of his profession, and became himself an expert. His most pronounced characteristic was his devotion to the ethics of his profession. He has been in poor health for several years, and was well advanced in years, and his death is not a great surprise. We shall miss him at the National gatherings. He was a good and useful man, and our profession is better because he honored it in a long life of devotion to its higher privileges.

